# Deep learning strategies for addressing issues with small datasets in 2D materials research: Microbial Corrosion

**DOI:** 10.3389/fmicb.2022.1059123

**Published:** 2022-12-22

**Authors:** Cody Allen, Shiva Aryal, Tuyen Do, Rishav Gautum, Md Mahmudul Hasan, Bharat K. Jasthi, Etienne Gnimpieba, Venkataramana Gadhamshetty

**Affiliations:** ^1^Department of Civil and Environmental Engineering, South Dakota Mines, Rapid City, SD, United States; ^2^Two-Dimensional Materials for Biofilm Engineering Science and Technology (2DBEST) Center, South Dakota Mines, Rapid City, SD, United States; ^3^Data-Driven Materials Discovery Center, South Dakota Mines, Rapid City, SD, United States; ^4^Department of Biomedical Engineering, University of South Dakota, Sioux Falls, SD, United States; ^5^Department of Materials and Metallurgical Engineering, South Dakota Mines, Rapid City, SD, United States

**Keywords:** 2D materials, coatings, graphene, hexagonal boron nitride, electrochemical impedance spectroscopy, machine learning, microbial induced corrosion

## Abstract

Protective coatings based on two dimensional materials such as graphene have gained traction for diverse applications. Their impermeability, inertness, excellent bonding with metals, and amenability to functionalization renders them as promising coatings for both abiotic and microbiologically influenced corrosion (MIC). Owing to the success of graphene coatings, the whole family of 2D materials, including hexagonal boron nitride and molybdenum disulphide are being screened to obtain other promising coatings. AI-based data-driven models can accelerate virtual screening of 2D coatings with desirable physical and chemical properties. However, lack of large experimental datasets renders training of classifiers difficult and often results in over-fitting. Generate large datasets for MIC resistance of 2D coatings is both complex and laborious. Deep learning data augmentation methods can alleviate this issue by generating synthetic electrochemical data that resembles the training data classes. Here, we investigated two different deep generative models, namely variation autoencoder (VAE) and generative adversarial network (GAN) for generating synthetic data for expanding small experimental datasets. Our model experimental system included few layered graphene over copper surfaces. The synthetic data generated using GAN displayed a greater neural network system performance (83-85% accuracy) than VAE generated synthetic data (78-80% accuracy). However, VAE data performed better (90% accuracy) than GAN data (84%-85% accuracy) when using XGBoost. Finally, we show that synthetic data based on VAE and GAN models can drive machine learning models for developing MIC resistant 2D coatings.

**Graphical Abstract:**
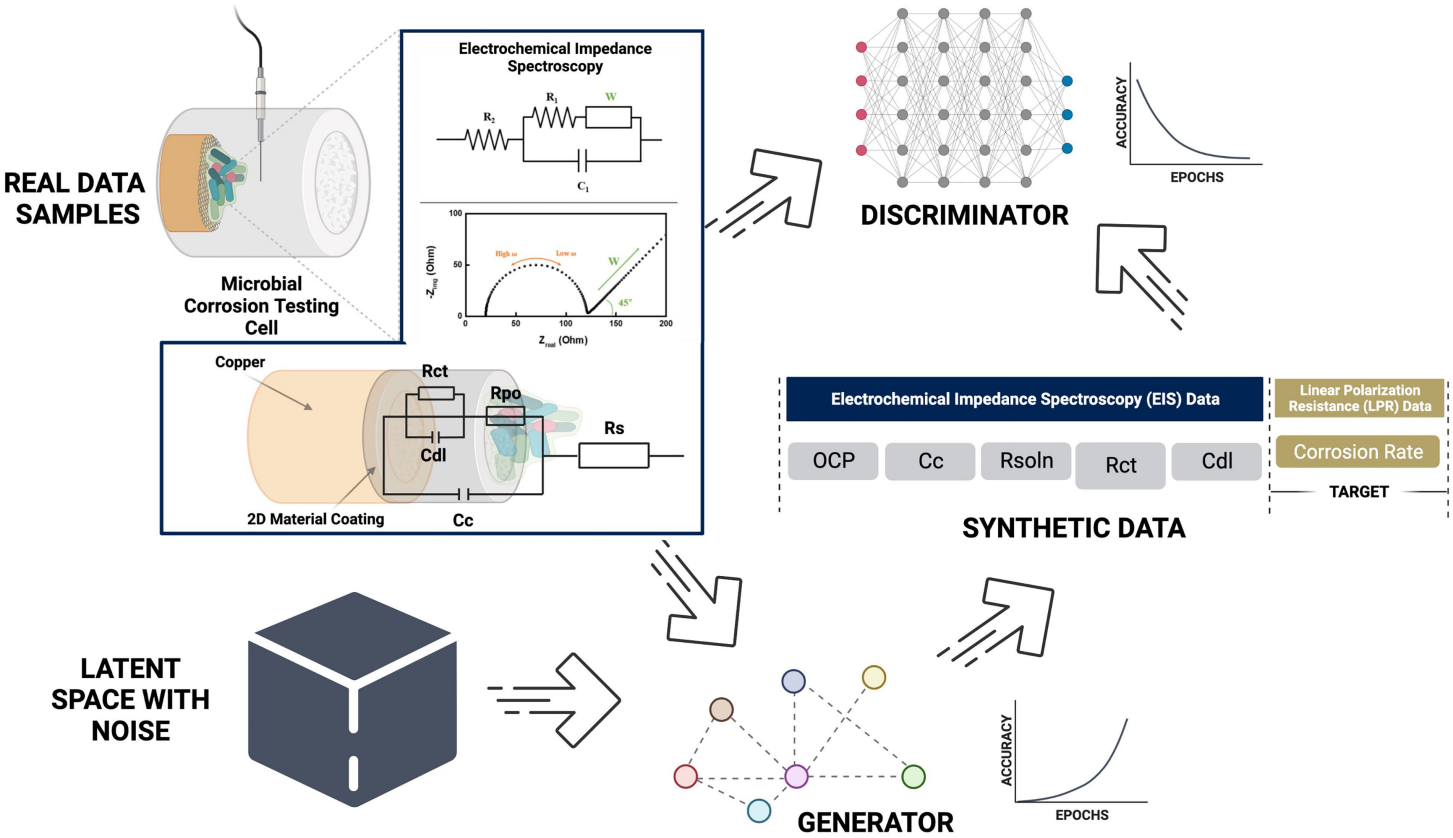
Created in biorender.

## 1. Introduction

Microbial induced corrosion (MIC) cause ~$30–50 billion of the annual expenditure (Heitz et al., 1996; Guo et al., 2018) in the oil and gas industry, marine infrastructure, water distribution systems, and many other environmental and energy sectors. The occurrence is unfortunately a spontaneous process that is unavoidable and can only be delayed, not prevented. Currently to delay this pipelines and holding tanks use solvent free epoxy liners, thiol monolayers, self-assembled monolayers and biocides to mitigate bacterial attachment and subsequent corrosion (Song and Feng, 2020). Recently, a new class of protective coatings based on 2D materials (e.g., graphene and hexagonal boron nitride) are being developed for MIC prevention applications (Chilkoor et al., 2019, 2020, 2021). This is due to their unique impermeability, inertness, excellent bonding, and passivation properties that resist the acts of corrosion. Unfortunately, other 2D materials are not providing the same promising results, many newly discovered 2D materials such as MoS_2_, NbSe_2_, and CrO_2_ have not been explored as extensively due to their structural instabilities in aggressive environments (Tanjil et al., 2019). To date empirical approaches have been the most common approach used in the development of protective coatings and is no longer sufficient (Wilson and Guan, 2020). Therefore, next generation methods based on machine learning (ML) need to be considered when developing next generation material coatings for microbial induced corrosion mitigation. Specifically, we look into how electrochemical datasets from corrosion experiments can be used to aid future ML models. ML has been used previously for corrosion detection (Galvāo et al., 2020; Xu et al., 2020; Diao et al., 2021; Coelho et al., 2022). A vast majority of all of these works use environment conditions and material composition as their selected input features. To our understanding there have been no ML papers reporting on microbial induced corrosion rates with the use of electrochemical circuit parameters.

Electrochemical reactions at a material solution interface can be broken down into a series of steps, including mass transport, charge transfer processes and adsorption. Methods such as electrochemical impedance spectroscopy (EIS) and linear polarization resistance (LPR) are used to generate these datasets. EIS is a rapid non-invasive technique widely applied to the analysis of conductive materials (Mansfeld, 1990). The EIS technique applies a frequency dependent sinusoidal input potential that leads to a current. The results are the detected as the changes in output potential and current. Because resistance is independent of frequency, and capacitance is inversely dependent to frequency, EIS measurements effectively differentiate between resistance and capacitance (Wang et al., 2019). By comparing the input values to the output values EIS is able to determine the impact, efficiency and magnitude of different components within the electrical circuit. The physical processes involved in electrochemical reactions are commonly represented in circuit elements. Understanding circuit elements provides information on kinetics, mass transport behavior and diffusion coefficients (Laschuk et al., 2019), providing surface coverage (Muthurasu and Ganesh, 2012), characterizing corrosion processes (Ramanathan and Fasmin, 2017), and determining the mechanisms of surface interactions with the electrode. Faradaic circuit components include Ionic (R_ion_) and electric (R_elec_) resistances that account for the ionic and electronic movement within the electrode, Bulk solution resistance (R_S_) which accounts for the resistance between the working and counter electrodes, and charge transfer resistance (R_CT_) which is the electron transfer resistance across the electrode-electrolyte interface. Non-faradaic components on the other hand are responsible for capacitance circuit elements and these include the double layer capacitance (C_dl_) that gives the specific capacitance at the interface of the electrolyte within the electrode, and the coating capacitance (C_c_) is the observed capacitance between the metal and electrolyte with the coating acting as the dielectric. While electrochemical circuit components are often used to estimate corrosion levels, there are no clear relationships between all electrochemical data and specific corrosion systems. The use of machine leaning (ML) algorithms may aid in the extraction of complex relationships from collected data.

Commonly machine learning models are trained with data sets ranging from tens of thousands to state of the art models on the order of millions of labels. When ML is applied to electrochemical data from corrosion studies these large datasets do not exist. In addition, we further decrease the dataset sizes with our criteria of electrochemical data from 2D materials used for microbial induced corrosion prevention. The small datasets are due to a few reasons: data generation from wet lab experiments is time-consuming and the use of 2D materials in microbial corrosive environments is in its infancy, as well as poor data sharing practices in literature. To increase our dataset sizes from our experimental work, we look into deep learning methods to improve our small datasets. This is the premise of data augmentation, where we quickly generate synthetic data to eliminate the time and efforts needed for wet lab experimentation. Data augmentation is a technique in which a training set is expanded with class-preserving transformations (Dao et al., 2019). There are two major families of deep generative models, variation autoencoder (VAE) and generative adversarial network (GAN). VAE’s have been used extensively in the fields of pathology detection (Uzunova et al., 2019), medical data (Pesteie et al., 2019), and image analysis (Biffi et al., 2019; Ahmad et al., 2022; Alves and Traina, 2022). Where GAN was been used in environmental monitoring (Wang et al., 2020), medical imaging Yi et al., 2019), and generation of synthetic test data for corroded pipelines (He and Zhou, 2022). The following are questions we aim to answer in the manuscript. (1) Can deep learning based data augmentation be used to generate statistically relevant electrochemical impedance parameters generated from small wet lab experimentation datasets. (2) Can synthetic data be paired with experimental data in machine learning models, XGBoost and neural networks, to accurately predict corrosion rate groupings. (3) Do larger electrochemical datasets generate insights, predictions, and or recommendations that were previously unavailable due to lack of relevant data. Here, we analyze synthetic data from VAE and GAN models for electrochemical modeling of microbial corrosive systems using electrochemical parameters.

## 2. Materials and methods

### 2.1. Data preparation

All experimental work was done using Desulfovibrio alaskensis strain G20 (DA-G20) that was anaerobically grown in the Lactate C (L-C) medium containing the following constituents (g/L): sodium lactate, 6.8; sodium sulfate, 4.5; sodium citrate, 0.3; dehydrated calcium chloride, 0.06; ammonium chloride, 1.0; magnesium sulfate, 2.0; potassium phosphate monobasic, 0.5 and yeast extract, 1.0. The listed L-C medium components were mixed thoroughly using type III ASTM Standards for Laboratory Reagent Water 3 (ASTM D1193-91). The pH of the medium was adjusted to 7.2 and then sterilized by autoclaving at 121 OC for 30 min. The DA-G20 cultures were grown in 150 ml serum bottles containing 100 ml of L-C having a headspace of N2-H2 (95% N2 v/v and 5% H2 v/v) (Qiu et al., 2011). DA-G20 cultures were incubated at 30°C using modest agitation (125 rpm) on an orbital platform shaker for 48 h.

To establish an electrochemical database for 2D materials we extracted 49 sets of EIS and LPR data, equivalent circuits and corresponding corrosion rates from current laboratory experimentation and published papers (Chilkoor et al., 2019, 2020, 2021). All data can be found in supplementary information. To observe how 2D materials increase corrosion resistance, corrosion rates were normalized to the bare metal samples ran in the same conditions. Therefore, samples with normalized corrosion rates less than 1, observed improve corrosion resistance, where rates larger than 1 observed decreases in corrosion resistance versus their bare metal controls. Data was then classified as effective coatings if normalized rates were less than 0.999, and a failed coating if higher than 1. From the classified corrosion resistances values seven input components (C_dl_, C_c_, m, OCP, R_soln_, R_ct_, and R_po_) were synthetically generated to match respective corrosion resistances. These parameters were chosen due to their importance in understanding physical processes during corrosion, such as, kinetics, mass transport and diffusion coefficients (Laschuk et al., 2019).

**Figure 1:**
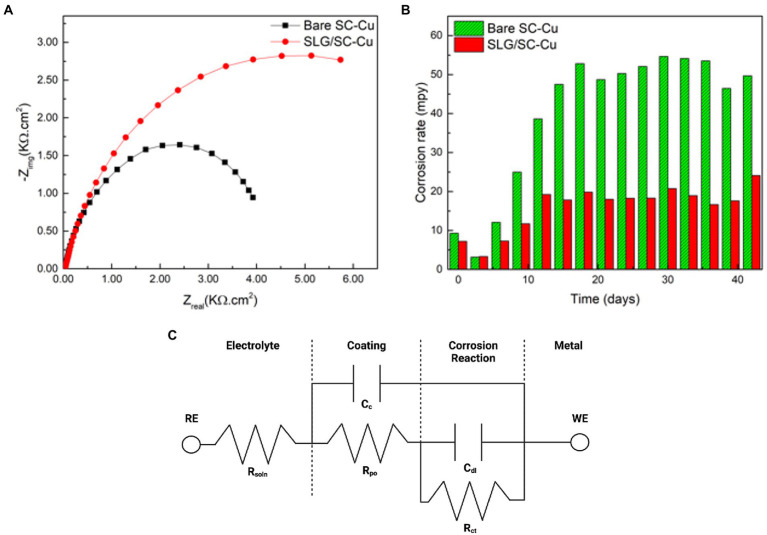
**(A)** Nyquist plot from electrochemical impedance spectroscopy. **(B)** Corrosion rates from linear polarization resistance. **(C)** equivalent circuit model derived from spectra.

All electrochemical data was collected at discrete timepoints. Therefore, when the electrochemical impedance parameters were used as labels for the supervised learning model, the corrosion rate target were continuous variables. While continuous variables are easy to relate to it is difficult from a predictive modeling point of view. Due to our small dataset the target corrosion rate variables were binned, meaning that the continuous variables were divided into two groupings, effective coatings (less than 0.999) and failed coatings (greater than 1.000), making it easier to discover patterns. The seven labels remained as continuous variables (Figure 1).

### 2.2. Data augmentation

To increase the number of training samples, variation autoencoder (VAE) and generative adversarial network (GAN) were used to generate synthetic samples for each class of corrosion resistances. First synthetic data was generated based on discrete corrosion rates, but provided worse results than when classified by effective coatings (less than 0.999) and failed coatings (greater than 1.000). 100 synthetic data points were added to the original 49 data points collected *via* experimentation and literature. Fifty of the samples were effective in coating resistance (encoded as 0), 50 of the samples were failed in coating resistance (encoded as 1). Corresponding equivalent circuit parameters were generated with respect to the coating resistance classification.

### 2.3. Machine learning models

**Figure 2:**
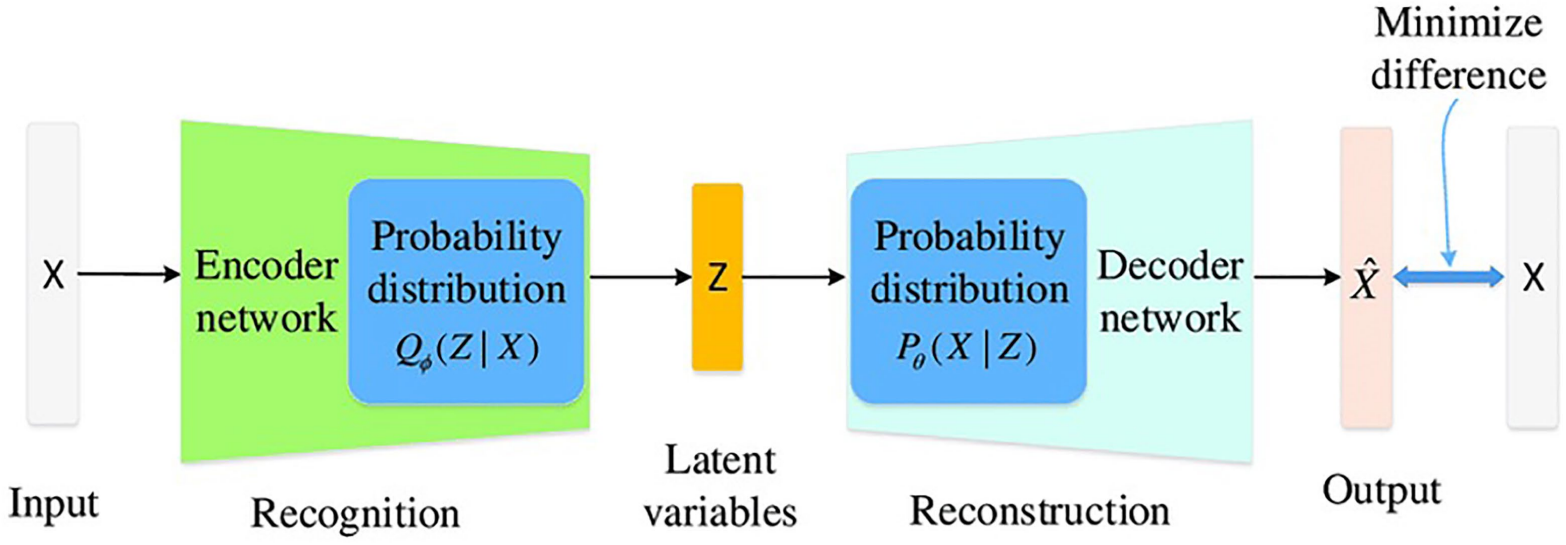
Generate tabular synthetic data using GAN architect. (Improving the Classification Effectiveness of Intrusion Detection by Using Improved Conditional Variational AutoEncoder and Deep Neural Network).

Electrochemical reactions at a material solution interface can be broken down into a series of steps, including mass transport, charge transfer processes and adsorption. EIS is able to determine the impact, efficiency and magnitude of different components within the electrical circuit. The physical processes involved in electrochemical reactions are commonly represented in these circuit elements. Understanding circuit elements provides information on kinetics, mass transport behavior and diffusion coefficients (Laschuk et al., 2019), providing surface coverage (Muthurasu and Ganesh, 2012), characterizing corrosion processes (Ramanathan and Fasmin, 2017), and determining the mechanisms of surface interactions with the electrode. EIS spectra are commonly represented as a Nyquist or Bode plot. A Nyquist plot represents the mass transfer and kinetic behavior, while the Bode plot represents frequency dependent behavior. Nyquist plots represent a combination of resistances, capacitances or inductances, and faradaic impedances. Faradaic circuit components include ionic (R_ion_) and electric (R_elec_) resistances that account for the ionic and electronic movement within the electrode, Bulk solution resistance (R_soln_) which accounts for the resistance between the working and counter electrodes, and charge transfer resistance (R_ct_) which is the electron transfer resistance across the electrode-electrolyte interface. Non-faradaic on the other hand is responsible for capacitance circuit elements and these include the double layer capacitance (C_dl_) that gives the specific capacitance at the interface of the electrolyte within the electrode. The coating capacitance (C_c_) is the capacitance of the coating that is covering the substrate. These circuit elements help describe the presence and magnitude of the corrosion process (Ahmad et al., 2021). Deep Neural Network with back propagation and XGBoost were performed in Python using Keras and Scikit Learn to verify the data generated from the EIS model. Seven components (C_dl_, C_c_, m, OCP, R_soln_, R_ct_, and R_po_) were chosen as the features of the dataset and were used as input for the machine learning models (Figure 2). Normalized corrosion rates were chosen as the model output. 1,000 pairs of inputs and outputs obtained at different timeframes were fed into the model with 75% used as training data and 25% used as testing data.

## 3. Results and discussion

### 3.1. Data augmentation

**Figure 3:**
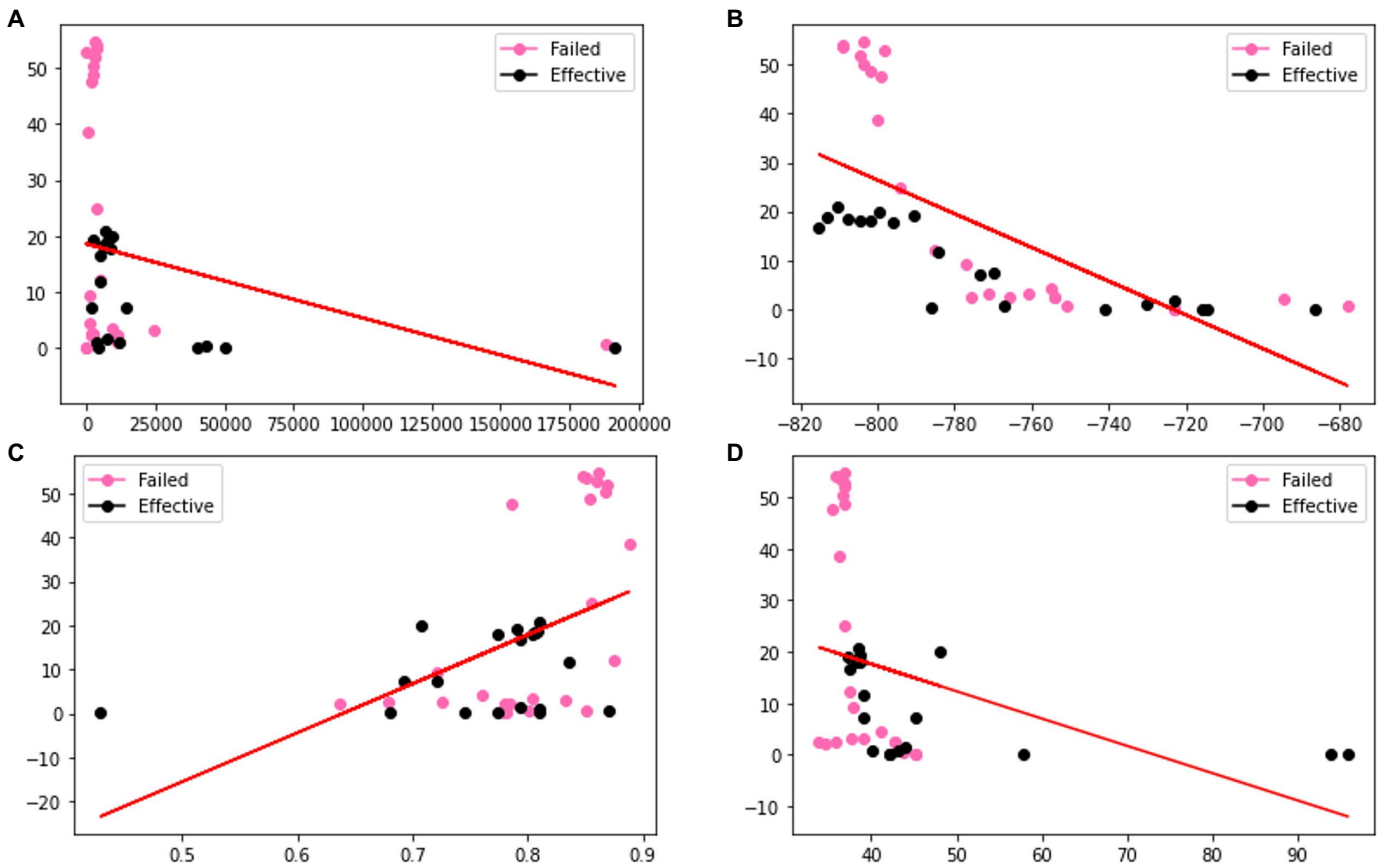
Visualization of the distributions of different columns **(A)** Rct **(B)** OCP **(C)** Rsoln **(D)** m of original dataset. Color coded by the label where pink is a failed coating (normalized corrosion rates greater than 1.000), and black is an effective coating (normalized corrosion rates under 0.999).

**Figure 4:**
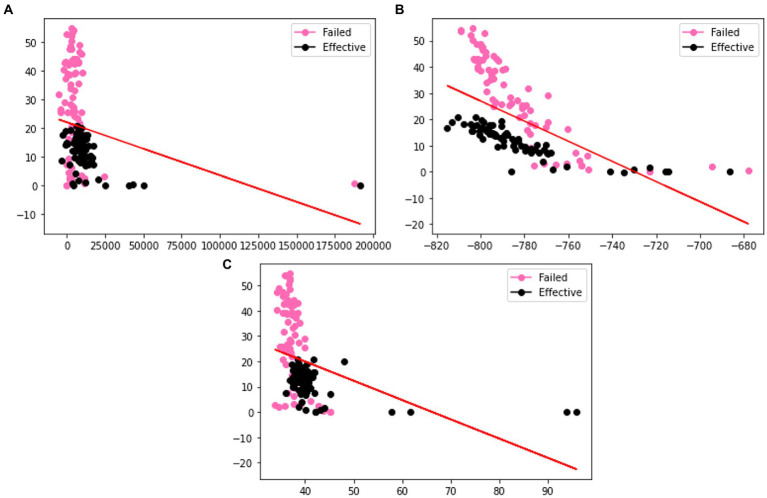
Visualization of the distributions of different columns **(A)** Rct **(B)** OCP **(C)** Rsoln of VAE generated dataset. Color coded by the label where pink is a failed coating (normalized corrosion rates greater than 1.000), and black is an effective coating (normalized corrosion rates under 0.999).

**Figure 5:**
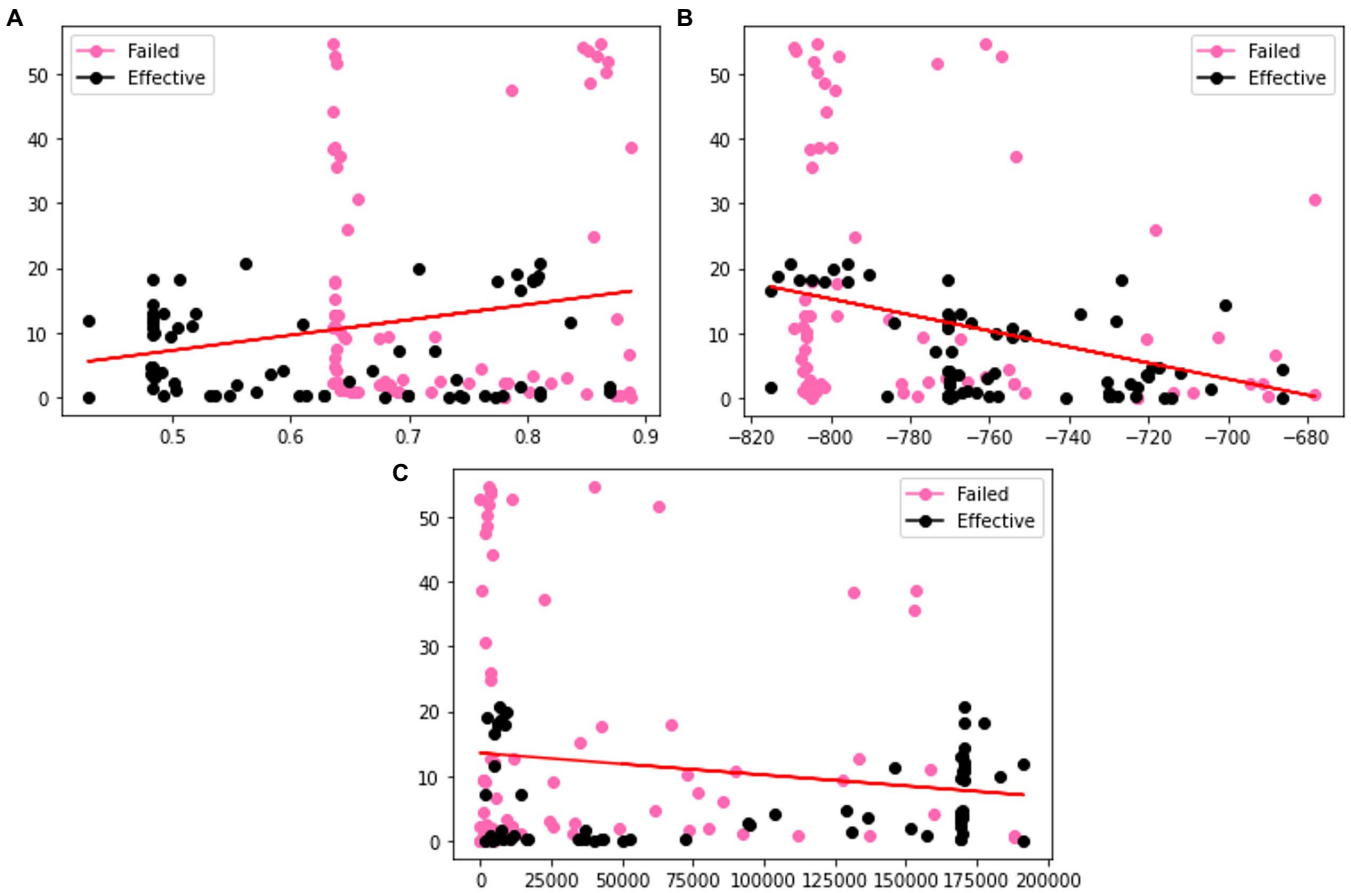
Visualization of the distributions of different columns **(A)** m **(B)** OCP **(C)** Rct of GAN generated dataset. Color coded by the label where pink is a failed coating (normalized corrosion rates greater than 1.000), and black is an effective coating (normalized corrosion rates under 0.999).

In order to verify the effectiveness of the augmentation, we visualize the original charge transfer resistance (Rct), open circuit potential (OCP) and solution resistance (Rsoln) data and the new samples generated by VAE and GAN augmentation models. Tables 1–3 show the statistics of the experimentation corrosion data and the synthetic corrosion data generated from GAN and VAE models. Figure 3 shows the original data generated doing wet lab experimentation. Figures 4, 5 show the distribution of original and synthetic data points using GAN and VAE models.

**Table 1:** Characteristics of the real corrosion data used in machine learning model.

Index	OCP	R_soln_	R_ct_	R_po_	C_c_	m	C_dl_
Max	−678.00	95.87	1.91 × 10^5^	1.65 × 10^5^	1.39 × 10^−3^	0.89	2.49 × 10^−2^
Min	−815.10	33.88	4.27 × 10^−1^	1.34 × 10^−1^	8.00 × 10^−6^	0.43	2.50 × 10^−11^
Mean	−770.90	42.30	1.66 × 10^4^	8.59 × 10^3^	4.21 × 10^−4^	0.79	1.22 × 10^−3^
Std	37.32	12.38	3.98 × 10^4^	2.94 × 10^4^	2.46 × 10^−4^	0.08	4.12 × 10^−3^

**Table 2:** Characteristics of the VAE based synthetic corrosion data used in machine learning model.

Index	OCP	R_soln_	R_ct_	R_po_	C_c_	m	C_dl_
Max	−734.56	61.73	2.48E+04	2.50E+04	7.62E-04	0.86	2.18E-03
Min	−804.21	34.13	5.11E+03	6.48E+03	2.29E-04	0.70	−7.33E-04
Mean	−787.26	38.50	6.40E+03	2.72E+03	4.63E-04	0.81	4.31E-04
Std	12.66	3.03	5.59E+03	4.12E+03	7.30E-05	0.03	5.70E-04

**Table 3:** Characteristics of the GAN based synthetic corrosion data used in machine learning model.

Index	OCP	R_soln_	R_ct_	R_po_	C_c_	m	C_dl_
Max	−678.00	95.87	1.92E+05	1.65E+05	1.39E-03	0.89	2.49 × 10^−2^
Min	−815.10	33.88	4.27E-01	1.37E-01	8.00E-06	0.43	2.50 × 10^−11^
Mean	−766.25	56.26	8.94E+04	5.13E+04	3.79E-04	0.62	2.79 × 10^−3^
Std	36.75	24.22	6.94E+04	3.99E+04	4.01E-04	0.11	5.78 × 10^−3^

### 3.2. Model training and testing

As the solution for quantifying important electrochemical parameter pertaining to microbial corrosion resistance using equivalent circuit components is not straightforward, we turned to machine learning to help leverage our growing database. eXtreme Gradient Boosting (XGBoost) and Neural Networks were tested in Python using Keras and Scikit learn and applied to check which model accurately classified the corrosion resistance data based on seven input variables (C_dl_, C_c_, m, OCP, R_soln_, R_ct_, and R_po_) and to verify if the data generated from the experiment can be tested using machine learning model. The Neural Network gave 45–50% accuracy at predicting the output of corrosion resistance. XGBoost outperformed all the other models with 90–92% accuracy in classifying the data accurately.

#### 3.2.1. Neural network

Seven input parameters (C_dl_, C_c_, m, OCP, R_soln_, R_ct_, and R_po_) were fed into the first hidden layer of neural network consisting of 12 nodes. The output from first hidden layer was then fed into second hidden layer consisting of 8 nodes in order to improve training. The first and second layer both used ReLU activation function (Qiu et al., 2011). ReLU function utilizes maximizer operation and can be written as:


(1) f(x)=max{0,z}

The sigmoid function maps the output received from hidden layers between 0 to 1 or 1 to −1 and can be used as a predictive model. The model is represented by:


(2) $$f\left(x\right)=\frac{1}{1+e^{-x}}$$

**Figure 6:**
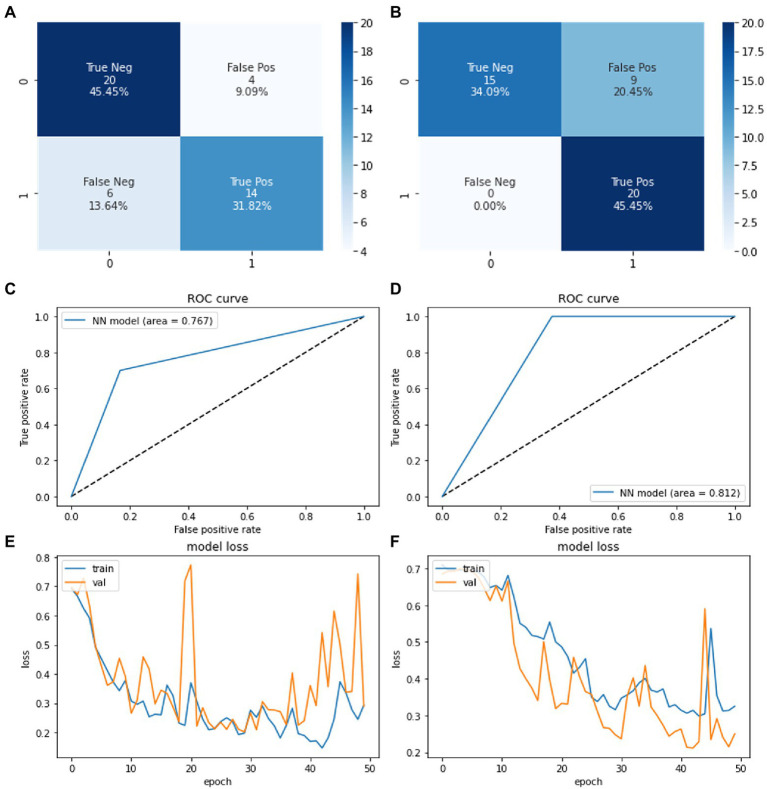
Confusion matrices showing the system performance of neural network **(A)** on VAE based synthetic data **(B)** on GAN based synthetic data. ROC curve showing the system performance of neural network model on **(C)** VAE generated synthetic data **(D)** GAN generated synthetic data. Training loss vs. epoch graph of neural network on **(E)** VAE based synthetic data **(F)** GAN based synthetic data.

The model was trained 50 times where a final accuracy of model was noted along with confusion matrix in Figure 6. The final accuracy of the model was calculated after implementing the testing dataset. Applying our Neural Network model on VAE augmented dataset, the training and evaluation accuracies obtained were 83.3 and 83.3% respectively, whereas when we applied k-fold cross validation, the testing accuracy obtained was 85.43% (±5.72%). Similarly, for GAN augmented dataset, the training and evaluation accuracies obtained were 86.11 and 88.9%, respectively, and the k-fold cross validation testing accuracy was 81.57% (±13.89%).

#### 3.2.2. XGBoost

**Figure 7:**
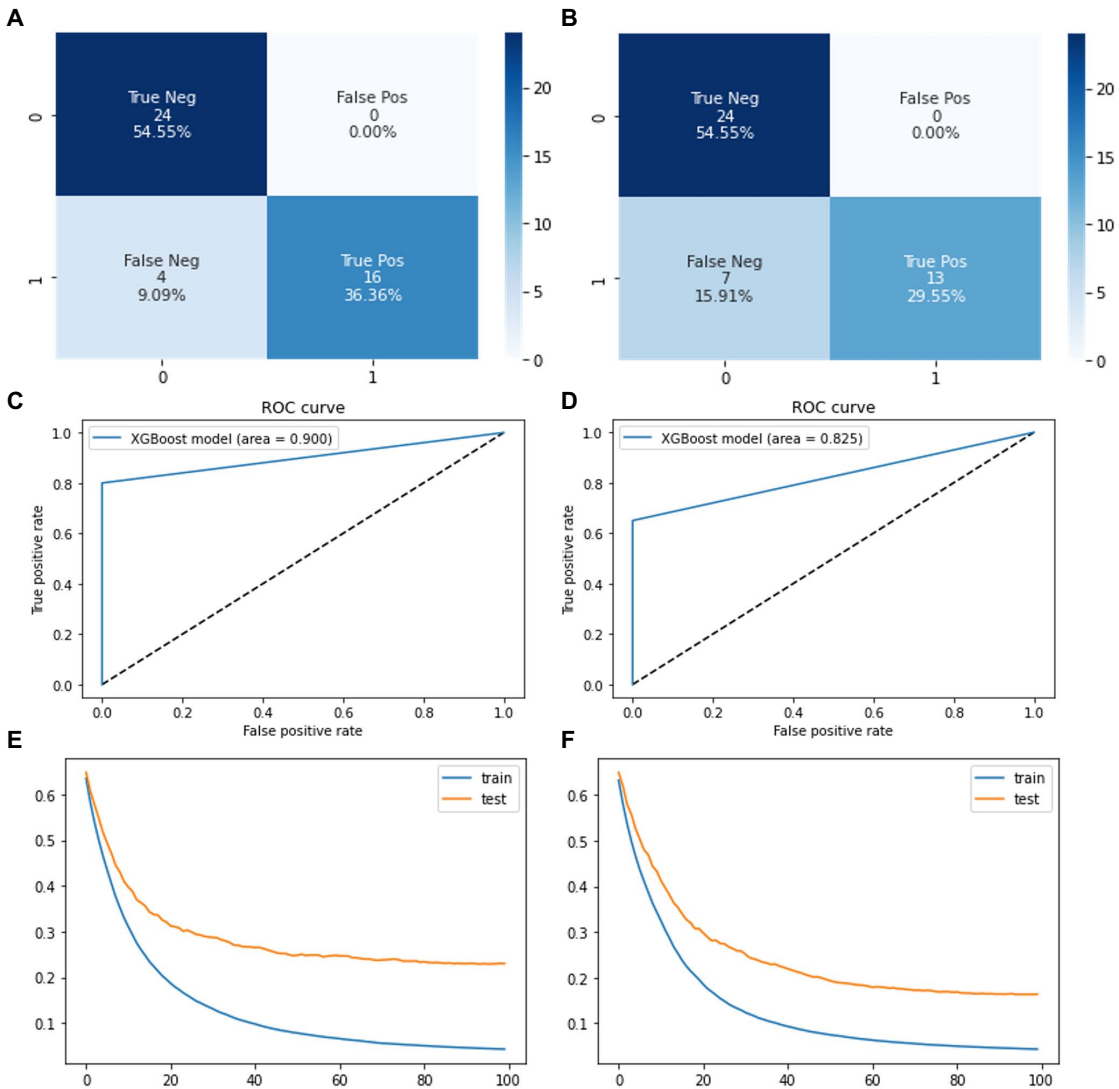
Confusion matrices showing the system performance of XGBoost on **(A)** VAE based synthetic data **(B)** GAN based synthetic data. ROC curve of XGBoost model on **(C)** VAE based synthetic data **(D)** GAN based synthetic data. Loss function for train and test set of XGBoost on **(E)** VAE based synthetic data **(F)** GAN based synthetic data.

The seven input parameters were fed into the XGBoost model to predict corrosion resistance classification. The model is built from XGBClassifier object of XGBoost python package. Gradient Boosting algorithm is the implemented form for XGBoost model. The model was trained and tuned using the training dataset of synthetic data from both VAE and GAN models. The model was trained for 100 times and final accuracy of model was noted along with confusion matrix in Figure 7. The final accuracy of the model was calculated after implementing the testing dataset.

XGBoost model loss function (Figures 7E,F) represents how well the model’s predictions fit the training data. Here, we find that XGBoost model is the most accurate model in predicting the corrosion resistance of 2D materials. Meaning that input impedance parameters can be used to accurately predict if 2D material coatings are effective or failed coatings to 90% accuracy. This information could increase accuracy of other corrosion models based on chemical and environmental conditions by introducing the accuracy generated from impedance parameters.

**Figure 8:**
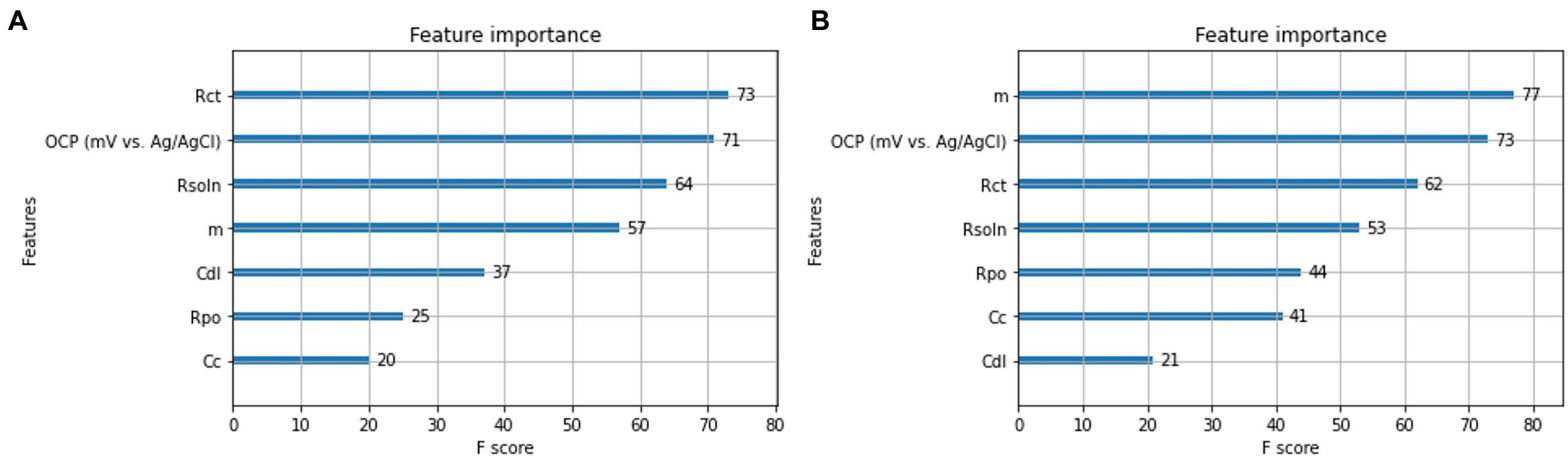
Feature component analysis using XGBoost. Of **(A)** VAE based synthetic data **(B)** GAN based synthetic data.

Figure 8 shows that the open circuit potential (OCP) and charge transfer resistance (R_ct_) are the two most important features for accurate corrosion resistance prediction. OCP is widely known in corrosion research as having a strong correlation with corrosion. This is because materials with a naturally high corrosion potential, meaning an increased corrosion is expected (Bastos et al., 2004). The charge transfer resistance (Rct) is a function for the electrochemical corrosion reactions intensity at coating/metal interface. The higher value of (Rct) implies the higher integrity of the coating system and then the slower development of corrosion reactions under the coatings. Knowing these input feature are the most important feature in predicting corrosion resistance for 2D material coatings implies our augmented data is in line with experimental and theoretical work.

### 3.3. Generalizability of machine learning models

Typical corrosion rate prediction models use chemical compositions as the input feature, and therefore have been limited to certain metals such as steel, copper, and aluminum (Wei et al., 2020). Challenges arise when predicting specific forms of corrosion such as atmospheric, marine and microbial induced due to their limited generalizability. The use of electrochemical impedance parameters as input functions in this study was firstly applied to generalize corrosion rate prediction. Meanwhile, the impedance features proposed an effective way to understand the influence of impedance components in an electrochemical system experiencing microbial induced corrosion. This approach has important application value in the future for guiding research into the use of impedance parameters for improved corrosion prediction models.

### 3.4. Challenges

In general, electrochemical studies always have some error while comparing the results obtained by different techniques and even by one technique measured on the equipment from different manufacturers. In the case of LPR and EIS, these techniques measure two different electrochemical parameters of the system. LPR provides real-time kinetics of the electrochemical processes. In opposite, EIS data is usually obtained at the OCP and provides measured values of the overall interfacial resistance at the electrode-electrolyte interface. Therefore, the prediction of corrosion rates derived from EIS impedance parameters adds a level of uncertainty. With larger datasets researchers will begin to understand trends within the electrochemical data and how it can be leveraged for many corrosion applications. Including, more accurate corrosion predictions models for specific environments and increased accuracy for 2D material development for specific applications.

## 4. Conclusion

In conclusion, we demonstrated a first-generation machine learning based electrochemical impedance spectroscopy model that predicts the corrosion resistance of 2D material coatings subjected to microbial induced corrosion. Data augmentation methods were used to increase the number of training samples to enhance neural networks and XGBoost algorithms feature representation. GAN synthetic data performed better in our neural network model up to 88.9%, while VAE models performed at 83.3%. Whereas VAE synthetic data performed better in our XGBoost model at 90.9% and GAN models performed at 84.1%. Experiment results show that augmented data can be used to increase algorithm performance. Prediction accuracy of 90.9% were observed using XGBoost. Note that our study is based on a small EIS sample set. Work will continue to be done to obtain additional EIS samples from different labs, including new 2D materials, environmental conditions and microbes. For more incorporation of machine learning within the corrosion community, efforts should be made to improve data sharing practices. Corrosion researchers would significantly benefit from increased access to high quality electrochemical datasets.

## Data availability statement

The original contributions presented in the study are included in the article/Supplementary material, further inquiries can be directed to the corresponding author.

## Author contributions

All authors listed have made a substantial, direct, and intellectual contribution to the work, and approved it for publication.

## Funding

This work was supported by the National Science Foundation/Experimental Program to Stimulate Competitive Research (EPSCoR) Grant OIA-1849206 awarded to Gilbert Ustad, and the Institutional Development Award (IDeA) from the National Institute of General Medical Sciences of the National Institutes of Health P20GM103443 (V.C. Huber).

## Conflict of interest

The authors declare that the research was conducted in the absence of any commercial or financial relationships that could be construed as a potential conflict of interest.

## Publisher’s note

All claims expressed in this article are solely those of the authors and do not necessarily represent those of their affiliated organizations, or those of the publisher, the editors and the reviewers. Any product that may be evaluated in this article, or claim that may be made by its manufacturer, is not guaranteed or endorsed by the publisher.
